# Tome's Dental Surgery

**Published:** 1859-10

**Authors:** 


*Tomes' Dental Surgery.*—The review, in another part of the present No. of the Journal, might seem to render a notice from us of Mr. Tomes' work unnecessary, but as we cannot endorse all the writer of the review says concerning the book, a brief expression of our own opinion of its merits is due to our readers

That the work is all the profession had a right to expect from a man occupying the position the author does, as a microscopist and teacher of dentistry in one of the Metropolitan hospitals of England, we dare not affirm, still, in our opinion, it constitutes a very creditatable contribution to the literature of the specialty to which the writer has, for a number of years, devoted a large portion of his time.

Nearly half of the treatise is devoted to teething. The author traces the progress of the teeth, from the first appearance of their rudiments as simple mucous papillæ, until the completion of the organs, describing, at the same time, the changes which take place in the maxillary bones. This, and some forty-eight pages, containing a description of the dental tissues, constitute the most valuable portions of the work, and although the author advances what we conceive to be some erroneous opinions with regard to the destruction of the roots of the temporary teeth, and the mechanism of the eruption of these organs, we, nevertheless, take pleasure in commending this part of the treatise, as containing the best summary of the subjects discussed, to be found in any other single work.

This portion of the book contains some engravings of curious and interesting abnormal development, both of the jaws and teeth. One of these represents a deformity of the lower jaw, occasioned by a cicatrix, consequent upon a burn, when the patient was only five years of age. The chin, in consequence, was drawn down near the sternum. The case is precisely like the one described by Dr. S. P. Hullihen some years ago in the Journal. The deformity in the case of Dr. Hullihen's patient, was completely removed by a series of most ingeniously contrived and skillfully executed surgical operations.

The diseases of the teeth and mouth, and practical dentistry, do not seem to have engaged as much of the author's attention as the physiological portions of his work. His descriptions are too brief to serve as a sufficient guide to the student and inexperienced dentist. They embody only a very condensed summary of the principal methods of operating pursued by dentists of the present day. But he very properly gives more prominence to caries of the teeth and its treatment, than to any of the other pathological conditions of these organs or of the mouth.

Among the materials recommended by Mr. Tomes for filling teeth, we notice *amalgam*—a compound, the use of which has long been repudiated by most really skillful operators in the United States. Any tooth that can be permanently preserved, and be retained with impunity in the mouth, can be filled with gold. The performance of the operation, in many instances, it is true, will require hours of tedious labor, and a degree of manual dexterity and correct judgment which only those who have determined to master the difficulties of such operations, possess.

But the labor required to make a difficult operation is no excuse for the use of a material so objectionable as the one under consideration, and we are sorry to see it recommended in so respectable a work.

The book would be read with more pleasure and profit, if the subjects discussed were treated in separate chapters, or under distinct headings.

But notwithstanding the objectionable things to which we have referred, the work is not without value, and should be read by every dentist.

